# Letter to Editor

**DOI:** 10.4274/tjod.galenos.2019.93585

**Published:** 2020-02-28

**Authors:** Demet Aydoğan Kırmızı, Taylan Onat, Emre Başer, Melike Demir Çaltekin

**Affiliations:** 1 Bozok Universty Faculty of Medicine, Obstetrics and Gynecology Department, Yozgat, Turkey

**Keywords:** Neutrophil/lymphocyte ratio, platelet/lymphocyte ratio, obstetric

## To the Editor,

We read the article of Jaffar DW. and Rabie MAF. titled “Maternal platelet-to-lymphocyte ratio at delivery can predict poor neonatal outcome in preterm births” published in 15(4)p.254,2018. In recent years, intensive research has been conducted on the use of hematologic inflammatory parameters such as the neutrophil/lymphocyte ratio and the platelet/lymphocyte ratio (PLR) in obstetric and gynecologic pathologies. On the other hand, there are few studies showing the effect of these parameters on neonatal outcomes. Therefore, we think that the results of this study are very important. PLR is associated with maternal immune activation and is therefore thought to increase more in inflammation-related processes such as pre-eclampsia and preterm labor^(1)^. In the materials and methods section of this study, we observed that the obstetric characteristics of the pregnant women group were not specified. It is not stated whether any of the 439 preterm labor cases in the study included early membrane rupture. It is also not indicated whether some subjects were excluded due to pregnancy-induced hypertension or pre-eclampsia. It is known that pre-eclampsia produces a maternal systemic inflammatory response, neutrophils increase and lymphocytes decrease^(2)^. Similarly, inflammation has been implicated in the etiology of early membrane rupture cases^(3)^. In the literature, the results studies evaluating the effect of PLR on neonatal outcomes vary^(4,5)^. The variable results can be attributed to maternal obstetric conditions (such as preterm labor, pre-eclampsia) and the different source of the blood sample (maternal/fetal). For these reasons, the diagnosis of patients, their hospitalization, status of thrombocytopenia due to pregnancy, and medical treatments should be specified. It should also be explained by which indication, in what manner, the number of gestational weeks at which they gave birth, and when the hemogram samples were taken. As can be expected, antibiotic, steroid applications, and developing obstetric complications may change the hemogram parameters. For these reasons, clarification of the materials and methods section of the article will provide a healthier evaluation of the results.
